# Transcranial doppler ultrasonography in neurocysticercosis

**DOI:** 10.1055/s-0044-1792017

**Published:** 2024-11-11

**Authors:** Hinpetch Daungsupawong, Viroj Wiwanitkit

**Affiliations:** 1 Vientiane College, Phonhong, Lao People's Democratic Republic.; 2 Saveetha University, Saveetha Institute of Medical and Technical Sciences, Saveetha Medical College and Hospital, Chennai, Tamil Nadu, India.

Dear Editor,

We hereby would like to comment on “Transcranial Doppler ultrasonography to evaluate cerebral hemodynamic changes in neurocysticercosis. 1 ” This study aimed to evaluate cerebral blood flow changes using transcranial Doppler echocardiography (TCD) in patients with subarachnoid cerebral cysts (SCC). A total of 53 patients – 29 with meningeal cysts and 24 with intracranial cysts – were evaluated at a referral hospital for neurological disorders. This evaluation consisted of a clinical interview, serum testing for intracranial cysts, and a TCD performed within two weeks of enrollment. Key hemodynamic measurements were recorded, such as mean flow velocity, peak systolic velocity, end diastolic velocity, and pulse index. The study found cerebral blood flow changes indicative of vasculitis in 12 patients, most of whom affected the middle cerebral artery, and there were significant gender differences between those with these changes.

One major flaw in this study is the small sample size, which may impair the accuracy of the results and their generalizability to a larger population. Although the study found a significant link between female gender and the presence of ultrasound-guided vasculitis, the small number of affected patients (12 out of 53) may have influenced the outcomes of these studies. Furthermore, the absence of statistical significance in the comparison of subcortical and subcortical tissue groups suggests that the study was underpowered to identify potential variations in cerebral hemodynamic alterations between the two types of NCC.

Future study should include bigger multicenter studies to enhance findings and uncover additional risk factors for cerebral hemodynamic abnormalities in NCC. Furthermore, combining modern neuroimaging techniques or biomarkers with TCD may provide a more complete picture of brain involvement in NCC and better knowledge of the underlying pathophysiology. Investigating the temporal link between cerebral hemodynamic alterations and clinical outcomes in NCC patients could also yield significant information.

The study's impact can be increased by including an element of novelty. For example, studying the effect of treatment technique on brain hemodynamic changes over time could be advantageous. Furthermore, using a longitudinal strategy to investigate how cerebral hemodynamic changes correlate with clinical symptoms and recovery may provide a better understanding of disease dynamics. Other demographic and clinical characteristics, such as past problems or cervical cyst therapy, may also yield useful information. It may also reveal vital information about the variability of cerebral hemodynamic response in NCC patients.
